# Checklist of the *Clubiona japonica*-group spiders, with the description of a new species from China (Araneae, Clubionidae)

**DOI:** 10.3897/zookeys.715.14645

**Published:** 2017-11-09

**Authors:** Hao Yu, Jianshuang Zhang, Jian Chen

**Affiliations:** 1 College of Chemistry and Life Sciences, Integrated Mountain Research Institute, Guizhou Education University, Guiyang, Guizhou, China; 2 School of Life Sciences, Guizhou Normal University, Guiyang, Guizhou, China; 3 Centre for Behavioural Ecology and Evolution (CBEE), College of Life Sciences, Hubei University, Wuhan, Hubei, China

**Keywords:** catalogue, *Japoniona*, Sac spiders, taxonomy

## Abstract

In the present paper, a worldwide checklist of *Clubiona japonica*-group spiders is provided based on published literature and authors’ collections. A new *japonica*-group species, *Clubiona grucollaris* **sp. n.** (♀♂) from Guizhou Province and Hainan Island of China is diagnosed, described, and illustrated. A distribution map of this species is given.

## Introduction

The genus *Clubiona* Latreille, 1804 contains 495 catalogued species and is widespread throughout most of the tropics and temperate regions of the world (World Spider Catalog 2017). Due to the high species diversity of *Clubiona*, several infrageneric classifications have been proposed by taxonomists, and therefore *Clubiona* species were assigned to a series of species-groups and/or subgenera (Simon 1932; Gertsch 1941; Lohmander 1944; Edwards 1958; Dondale and Redner 1976, 1982; Mikhailov 1990, 1991, 1995, 2002; Deeleman-Reinhold 2001; Wunderlich 2011).

*Japoniona* was established as a subgenus by Mikhailov (1990), including only one species-group: *japonica*-group. Later, the subgenus Japoniona was suppressed by Deeleman-Reinhold (2001) and reverted to *japonica* species-group. In the same book, Deeleman-Reinhold (2001) carried out intensive research on this group’s limits, supplemented some characters to support the monophyly of the group, and provided a checklist of *C. japonica*-group species from Southeast Asia. During the past decade, at least nine species belonging to the *japonica* species-group were reported and described from southeast Asia, China, and India (Dankittipakul and Singtripop 2008; Jäger and Dankittipakul 2010; Dankittipakul et al. 2012; Keswani and Vankhede 2014; Wu and Zhang 2014). However, a few other known species are not assigned, although they exhibit typical *japonica*-group features. The first goal of this paper is to provide a checklist as complete as possible of the current *japonica*-group species.

Various field collections in Guizhou Province, China were carried out by the colleagues of Hubei University in 2014 and 2016. Four males and 20 females were collected in these field explorations, among which one pair were captured during mating; thus, they are conspecific. Additionally, one male collected from Hainan Island was examined, and no differences from the Guizhou specimens were observed. All specimens possess certain characters associated with the *japonica*-group, but can be easily distinguished from the other *japonica*-group species. This species is new to science and is described under the name of *Clubiona grucollaris* sp. n.

## Materials and methods

The checklist is based on an examination of specimens deposited in the "Centre for Behavioural Ecology and Evolution" (CBEE) and reviews of the published literature, including several recent world catalogues of spiders (Lin and Li 2016; World Spider Catalog 2017).

Spiders were fixed and preserved in 80% ethanol. Specimens were examined with an Olympus SZX7 stereomicroscope; details were studied with an Olympus BX51 compound microscope. Male palps and female epigynes were examined and illustrated after being dissected. Spermathecae were cleared in boiling KOH solution to dissolve soft tissues. Photos were made with a Cannon EOS70D digital camera mounted on an Olympus BX51 compound microscope. The digital images were taken and assembled using Helifocus 3.10 software package. The drawings were made using an Olympus drawing tube. Most of the hairs and macrosetae are not depicted in the palp and epigyne images.

All measurements were obtained using an Olympus SZX7 stereomicroscope and given in millimetres. Eye diameters are taken at widest point. The total body length does not include chelicerae or spinnerets length. Leg lengths are given as total length (femur, patella + tibia, metatarsus, tarsus). The type specimens of the new species are deposited in College of Chemistry and Life Sciences, Guizhou Education University, Guiyang, Guizhou, China

Abbreviations used are:

**A** epigynal atrium;

**AER** anterior eye row;

**ALE** anterior lateral eyes;

**AM** atrial margin;

**AME** anterior median eyes;

**AME–AME** distance between AMEs;

**AME–ALE** distance between AME and ALE;

**BS** bursa;

**C** conductor;

**CD** copulatory duct;

**CO** copulatory opening;

**E** embolus;

**FD** fertilization duct;

**MOQ** median ocular quadrangle;

**MOQL** length of MOQ;

**MOQA** MOQ anterior width;

**MOQP** MOQ posterior width;

**PER** posterior eye row;

**PLE** posterior lateral eyes;

**PME** posterior median eyes;

**PME–PME** distance between PMEs;

**PME–PLE** distance between PME and PLE;

**RTA** retrolateral tibial apophysis;

**SB** spermathecal bases;

**SH** spermathecal heads;

**SP** spermatheca;

**SS** spermathecal stalks;

**TA** tegular apophysis.

The terminology used in text and figure legends follows Yu et al. (2012).

## Taxonomy

### Family Clubionidae Wanger, 1887

#### Genus *Clubiona* Latreille, 1804

##### The japonica

Taxon classificationAnimaliaAraneaeClubionidae

-group

###### Diagnosis.

**Figures 1–3.**
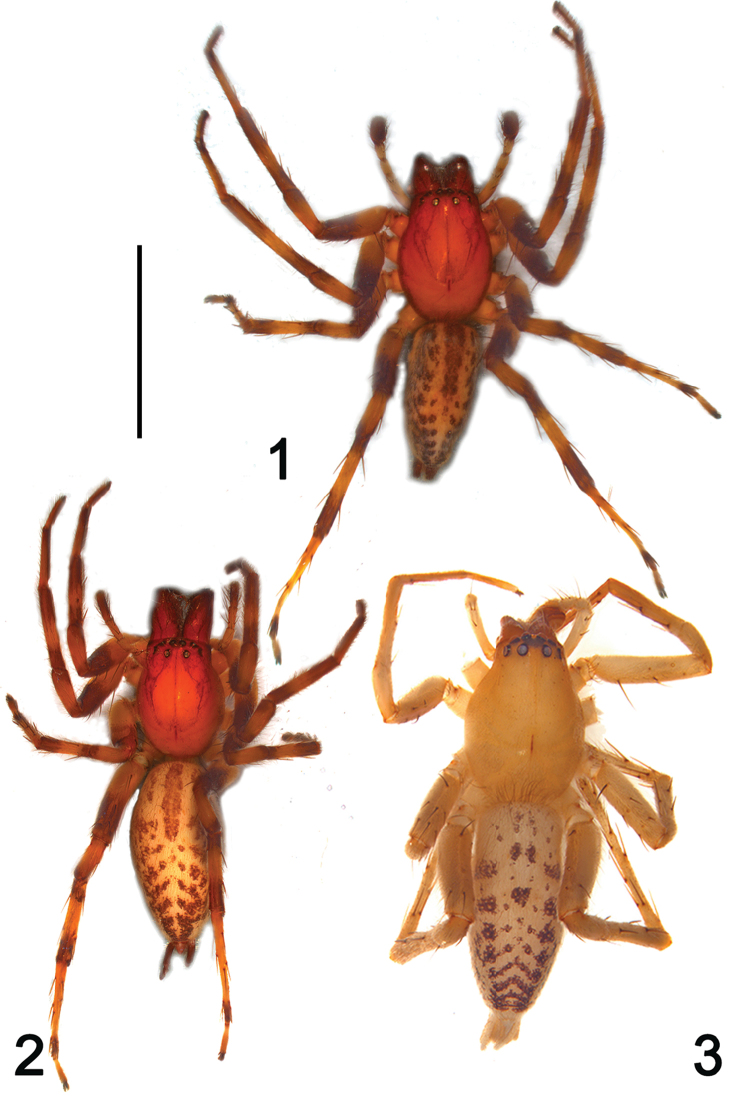
Habitus of *Clubiona grucollaris* sp. n. and *C. filicata* O. Pickard-Cambridge, 1874, dorsal view. **1** *C. grucollaris* sp. n., male holotype **2** *C. grucollaris* sp. n., female paratype **3** *C. filicata* O. Pickard-Cambridge, 1874, male from Guangxi, China. Scale bars 5 mm (**1–2**); 2.5 mm (**3**).

**Figures 4–5.**
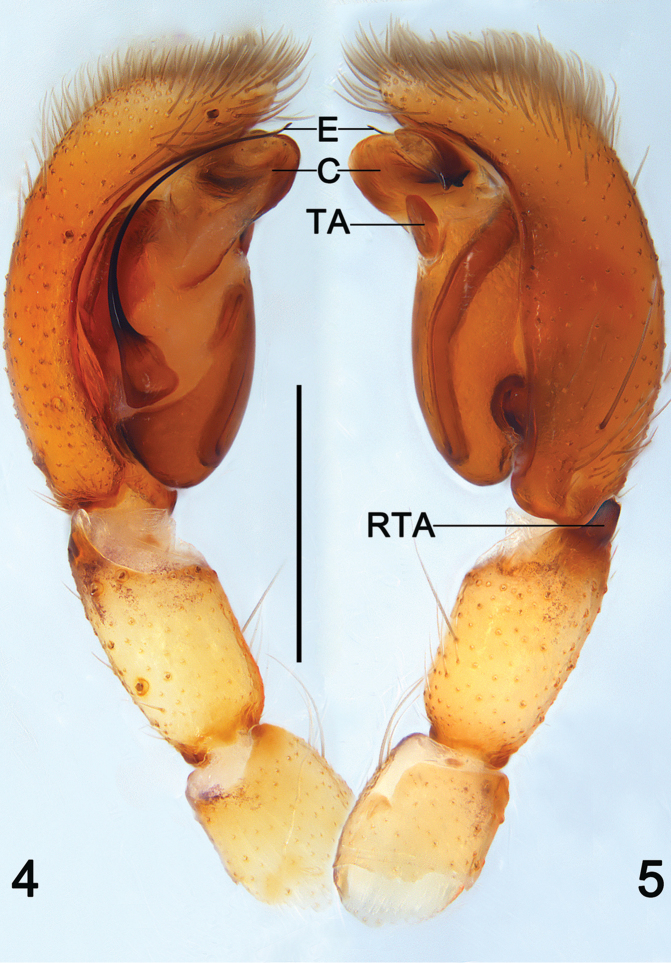
*Clubiona grucollaris* sp. n., male holotype. **4** left palp, prolateral view **5** same, retrolateral view. Scale bars 0.5 mm.

**Figures 6–8.**
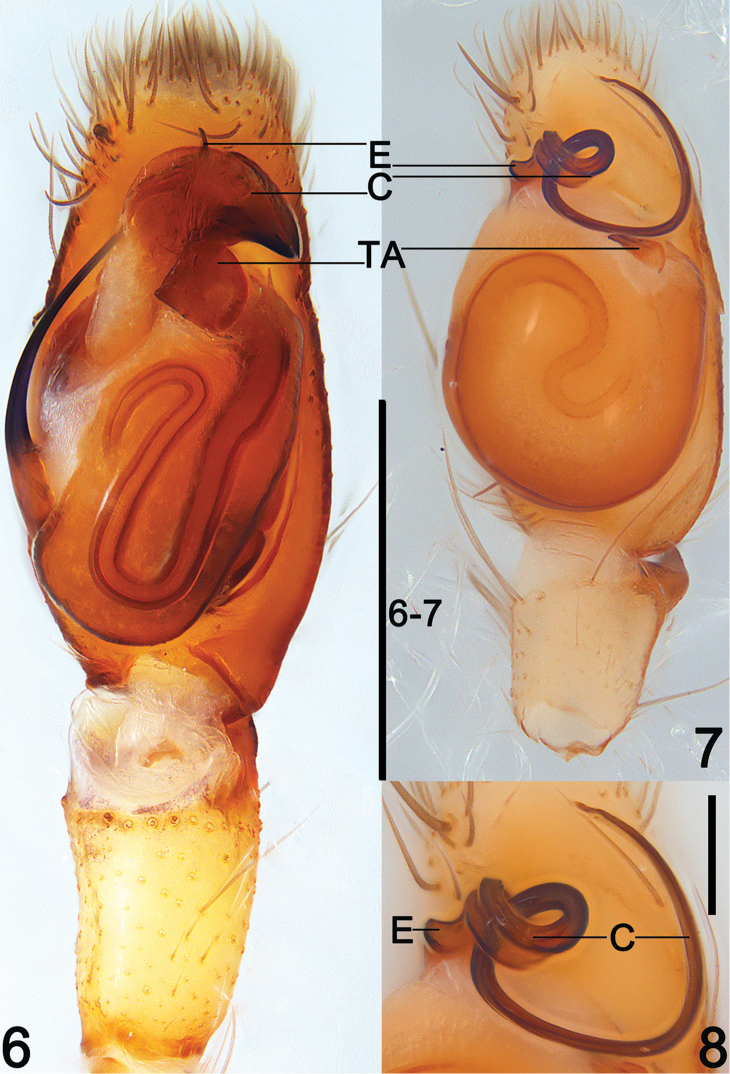
Left male palp of *Clubiona grucollaris* sp. n. and *C. filicata* O. Pickard-Cambridge, 1874, ventral view. **6** *C. grucollaris* sp. n., male holotype **7** *C. filicata* O. Pickard-Cambridge, 1874, male from Guangxi, China **8** *C. filicata* O. Pickard-Cambridge, 1874 from Guangxi, China, apical appendage of tegulum, ventral. Scale bars 0.5 mm (**6–7**); 0.1 mm (**8**).

**Figures 9–12.**
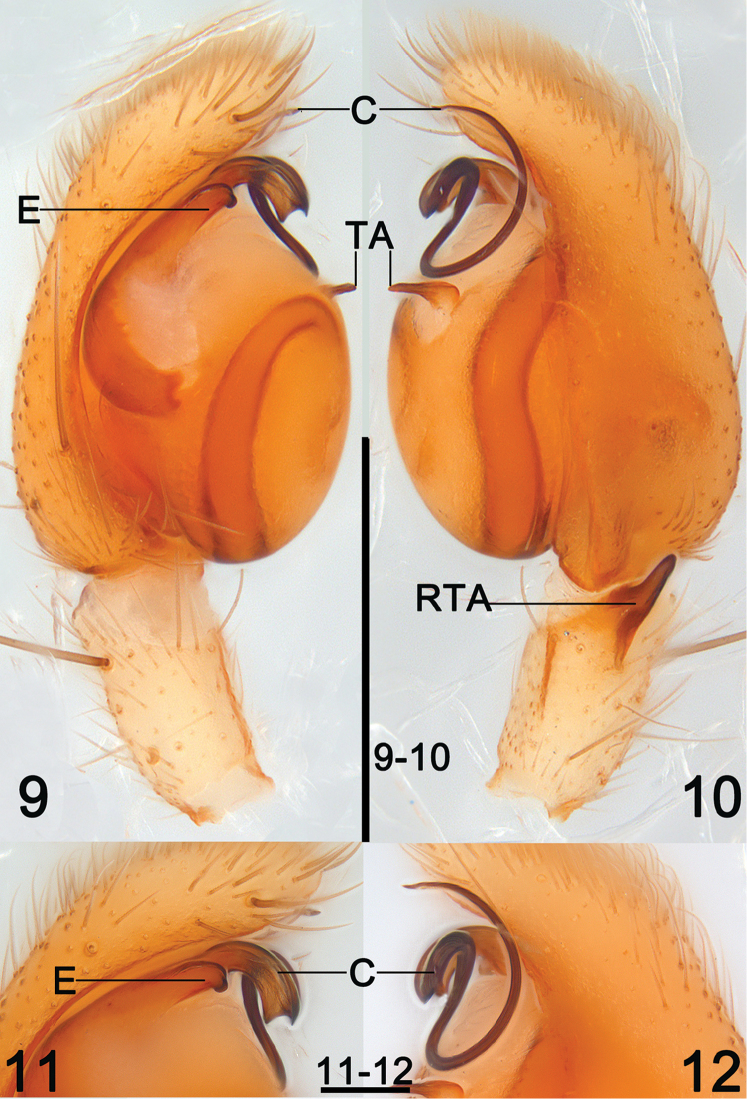
*Clubiona filicata* O. Pickard-Cambridge, 1874, male from Guangxi, China. **9** left palp, prolateral view **10** same, retrolateral view **11** apical appendage of tegulum, prolateral view **12** same, retrolateral view. Scale bars 0.5 mm (**9–10**); 0.1 mm (**11–12**).

**Figures 13–14.**
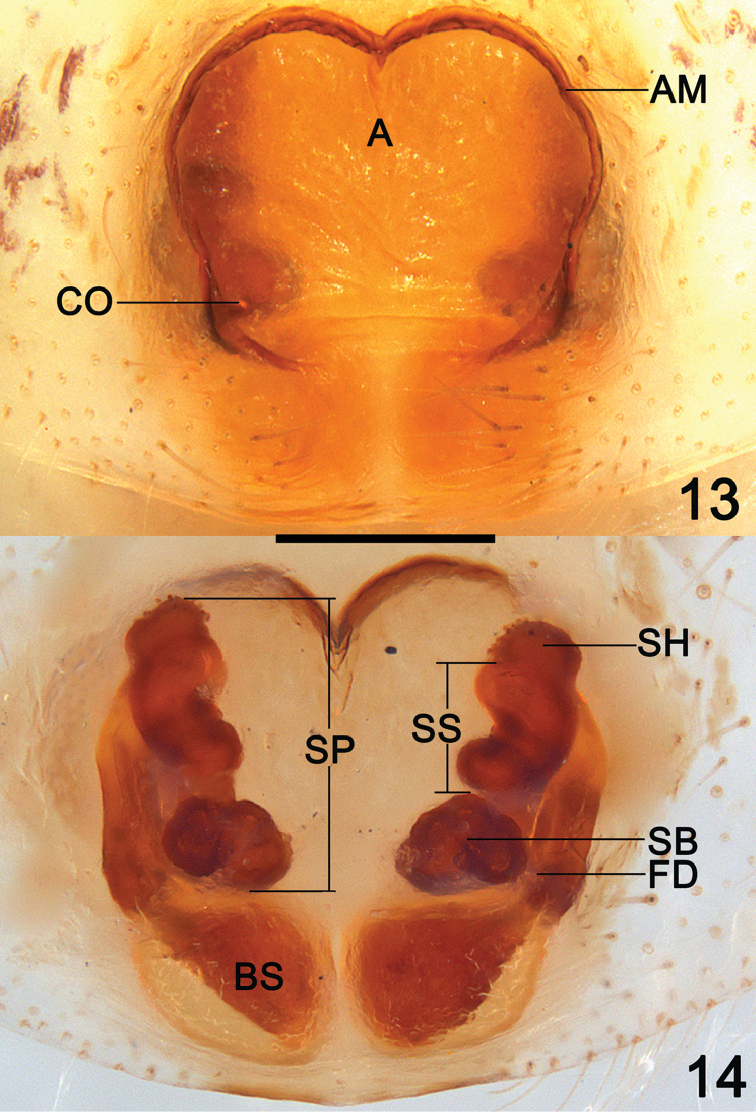
*Clubiona grucollaris* sp. n., female paratype. **13** epigyne, ventral view **14** vulva, dorsal view. Scale bars 0.2 mm.

In general, members of the *japonica*-group can be recognized by the following combination of characters (see also Dankittipakul and Singtripop 2008): dark colour pattern of carapace and dorsum of opisthosoma (Figs 1–3); the male retrolateral tibial apophysis small and not branched (Figs 5, 10), the sperm duct is sinuate and distinct (Figs 6–7), the embolus filiform or reduced (Figs 4–9, 11), the conductor sclerotized with variable shapes (e.g. a small tubercle in *C. picturata* Deeleman-Reinhold, 2001, long and filiform in *C. biembolata* Deeleman-Reinhold, 2001 and *C. filicata* O. Pickard-Cambridge, 1874, large and beak-shaped in *C. japonica* L. Koch, 1878 and *C. grucollaris* sp. n., Figs 4–12); the female epigyne has a relatively large atrium situated anteriorly, and the copulatory openings are located in rebordered groove of atrial margin (Fig. 13). The *japonica*-group resembles the *corticalis*-group in having the similar simple palp bulb in male, the atrium and copulatory openings located anteriorly in female, however, the latter can be distinguished from the former by: (1) the lack of a colour pattern on the opisthosoma; (2) the presence of a inflated tegulum with indistinct sperm duct; (3) the conductor membranous or absent; (4) the presence of a ventral tibial apophysis in many species; (5) the atrium is significantly smaller or absent; (6) copulatory openings are often located at anterior part of the epigynial plate, instead of close to the middle part in the *japonica*-group. All the provided *corticalis*-group characters are according to Deeleman-Reinhold (2001) and recent clubionid papers such as Wu and Zhang (2014) and Liu et al. (2016).

###### Taxonomic notes.

**Figures 15–19.**
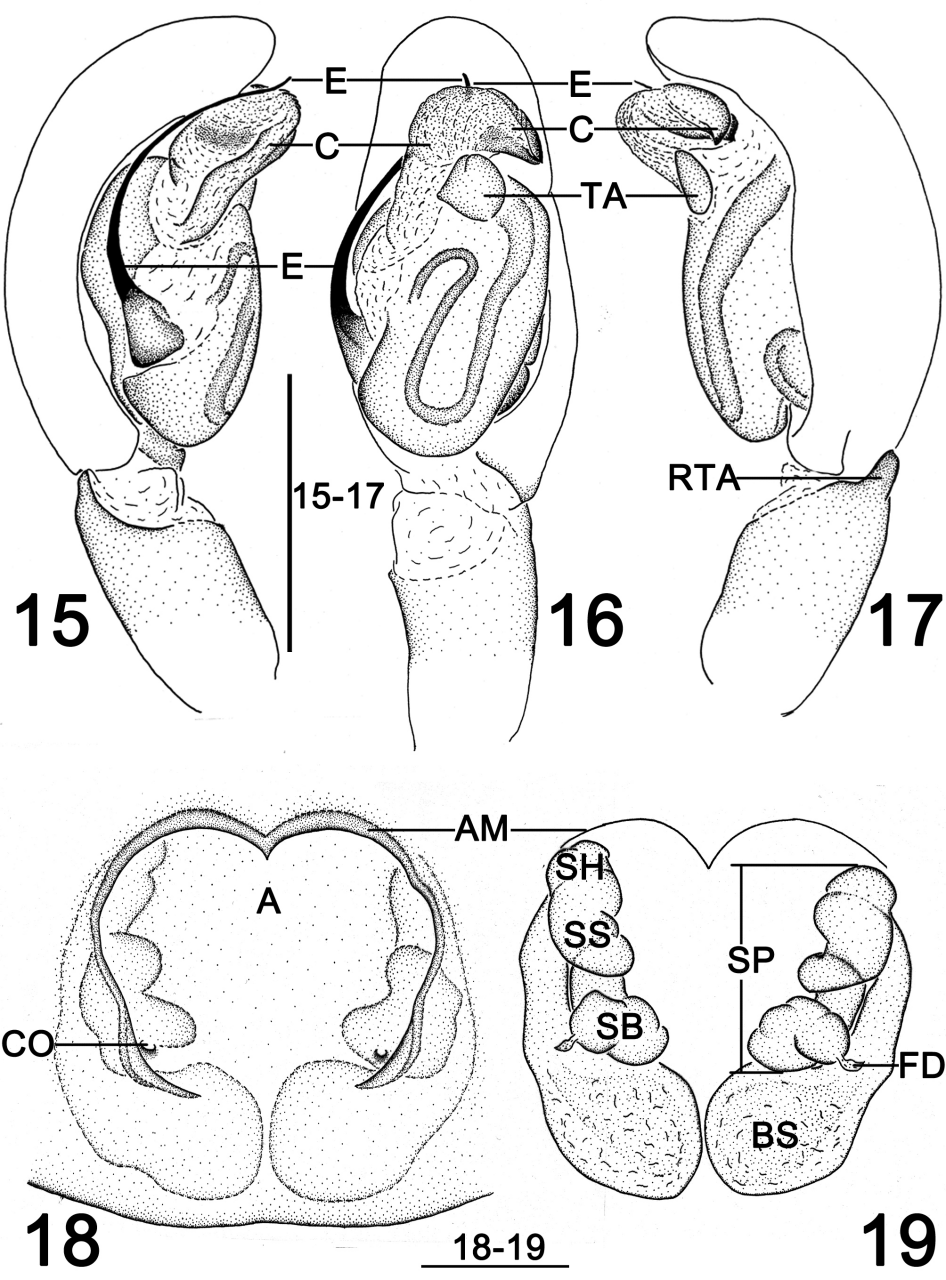
*Clubiona grucollaris* sp. n., male holotype and female paratype. **15** left palp, prolateral view **16** same, venteral view **17** same, retrolateral view **18** epigyne, ventral view **19** vulva, dorsal view. Scale bars 0.5 mm (**15–17**); 0.2 mm (**18–19**).

Dankittipakul and Singtripop (2008) divided the Southeast Asia *japonica*-group into two lineages. It appears that this standard of division may also apply to the *japonica*-group from China. The species of the 1^st^ lineage have a large sclerotized and beak-shaped conductor that aligned transversely on apical part of the bulb (Figs 4–6, 15–17), such as *C. circulata* Zhang & Yin, 1998, *C. calycina* Wu & Zhang, 2014 and *C. grucollaris* sp. n., etc. Members of the 2^nd^ lineage share the following characters: the reduced embolus; a long and filiform conductor; and the embolus and conductor fused with each other, forming an apical appendage together and situated on the apical portion of the tegulum (Figs 7–12). The 2^nd^ lineage includes *C. filicata* and *C. filoramula* Zhang & Yin, 1998.

In spite of the variable conductor in the male palp, the female genitalia of the two different lineages are very similar. The epigynial plate has a large atrium situated anteriorly, and the atrium is bounded by an atrial margin. The posterior atrial margins are often not rebordered. Copulatory openings relatively small, located in rebordered groove of basolateral atrial margin (Figs 13, 18). Vulva consisting of anterior spermathecae and posterior bursae. The bursae are membranous, larger than the spermathecae (Figs 14, 19).

Strictly based on the group characters, figures and text descriptions of 495 *Clubiona* species were checked one by one. In this work, we focused on ungrouped species, but also considered grouped species based on previous infrageneric revisions (Mikhailov1990, 1991, 1995, 2002; Deeleman-Reinhold 2001). As a result, there are at least 31 *japonica*-group species all over the world (but mainly distributed in Asia) at present, among which 9 species were recorded from China, including a new species described here as *Clubiona grucollaris* sp. n. (see Table 1).

**Table 1.** A list of current *Clubiona japonica*-group species in alphabetical order.

	Species name	Known sex	Distribution
1	*C. annuligera* Lessert, 1929	♂♀	Congo, Mozambique
2	*C. biembolata* Deeleman-Reinhold, 2001	♂♀	Borneo
3	*C. bilobata* Dhali, Roy, Saha & Raychaudhuri, 2016	♀	India
4	*C. calycina* Wu & Zhang, 2014	♂♀	China
5	*C. campylacantha* Dankittipakul, 2008	♂♀	Thailand
6	*C. charleneae* Barrion & Litsinger, 1995	♂♀	Philippines
7	*C. circulata* Zhang & Yin, 1998	♂♀	China
8	*C. coreana* Paik, 1990	♂♀	Russia, Korea, China
9	*C. digitata* Dankittipakul, 2012	♂♀	Thailand
10	*C. drassodes* O. Pickard-Cambridge, 1874	♂♀	India, Bangladesh, China
11	*C. filicata* O. Pickard-Cambridge, 1874	♂♀	India, Bangladesh, Pakistan, Thailand, Myanmar, Laos, China
12	*C. filifera* Dankittipakul, 2008	♂♀	Thailand
13	*C. filoramula* Zhang & Yin, 1998	♂	China
14	*C. foliata* Keswani & Vankhede, 2014	♂♀	India
15	*C. gallagheri* Barrion & Litsinger, 1995	♀	Indonesia
16	*C. japonica* L. Koch, 1878	♂♀	Russia, China, Korea, Japan
17	*C. lala* Jäger & Dankittipakul, 2010	♀	Laos
18	*C. melanosticta* Thorell, 1890	♂♀	Thailand, Sumatra, Krakatau, New Guinea
19	*C. melanothele* Thorell, 1895	♀	Myanmar, Thailand, Laos, Sumatra
20	*C. munda* Thorell, 1887	♀	Myanmar
21	*C. nigromaculosa* Blackwall, 1877	♂♀	Seychelles, Réunion
22	*C. octoginta* Dankittipakul, 2008	♂♀	Thailand
23	*C. picturata* Deeleman-Reinhold, 2001	♂♀	Bali
24	*C. pila* Dhali, Roy, Saha & Raychaudhuri, 2016	♀	India
25	*C. pupula* Thorell, 1897	♂♀	Myanmar
26	*C. scandens* Deeleman-Reinhold, 2001	♂♀	Borneo
27	*C. submaculata* (Thorell, 1891)	♂♀	Nicobar Is.
28	*C. suthepica* Dankittipakul, 2008	♂♀	Thailand
29	*C. vigil* Karsch, 1879	♂♀	Russia, Korea, Japan, China
30	*C. vukomi* Jäger & Dankittipakul, 2010	♂	Thailand, Laos
31	*C. grucollaris* sp. n.	♂♀	China

##### Clubiona grucollaris sp. n.

Taxon classificationAnimaliaAraneaeClubionidae

http://zoobank.org/25D9CD29-56E7-4D2E-9FBD-DB7C24DA5F1C

Figs 1–2 , 4–5 , 6 , 13–14 , 15–19 , 20

###### Type material.

**Holotype** ♂ (HUBU-GZ-IV-140057): China, Guizhou Province, Tongren City, Fanjing Mountain Nature Reserve (578 m; 21°51'12"N, 108°46'45"E), 3 August 2014, Jian Chen and Jianyong Li leg. Paratypes: 2 ♂ and 18 ♀, same data as holotype; 11 ♀, Tongren City, Mayanghe Nature Reserve (394 m; 28°46'53"N, 108°12'32"E), 15 August 2014, Mu Yan and Yaqian Fu leg; 1 ♂ and 1 ♀, Tongren City, Fanjing Mountain Nature Reserve (539 m; 27°50'42"N, 108°46'48"E), 6 April 2016, Hao Yu and Yang Zhong leg. 1 ♂, Hainan Province, Qiongzhong County, Limu Mountain Nature Reserve (417 m; 19°50'06"N, 109°47'52"E), 1 October 2009, Hao Yu and Zhenyu Jin leg.

###### Etymology.

The specific name is an adjective and is derived from the combination of two Latin words: *gru* (crane) + *collaris* (with neck), referring to the long and cylindrical conductor base, which is like the neck of crane.

###### Diagnosis.

*Clubiona grucollaris* sp. n. resembles the other *japonica*-group species by the similar habitus (Figs 1–3), but is consistently separable by its genitalia. Males of *Clubiona grucollaris* sp. n. appear to be closely related to *C. circulata* (Zhang and Yin 1998: 9, f. 1–3), *C. calycina* (Wu and Zhang 2014: 211, f. 1–12), and *C. suthepica* (Dankittipakul and Singtripop 2008: 42, f. 22–23, 56–58) in having the filiform embolus, and heavily sclerotized distal apex of beak-shaped conductor, but can be easily distinguished from these species by the crane’s neck-shaped conductor base, and by the nearly U-shaped sperm ducts (Figs 4–6, 15–17). Females of *C. grucollaris* sp. n. are similar to *C. circulata* (Zhang and Yin 1998: 9, f. 4–5), *C. filifera* (Dankittipakul et al. 2012: 57, f. 18–19, 23–24) and *C. octoginta* (Dankittipakul and Singtripop 2008: 39, f. 17–19, 45–46) by the broad atrium situated anteriorly, and the membranous bursae situated posteriorly, but can be recognized by the more or less inverted trapezoidal atrium with M-shaped anterior margin, and by the spiral spermathecae (Figs 13–14, 18–19).

###### Description.

***Male***. Total length 6.23–7.75. Holotype (Fig. 1): body 7.54 long; carapace 3.75 long, 2.42 wide; abdomen 3.96 long, 1.76 wide. *Carapace* brownish red, with a distinctive pattern on pars cephalica consisting of a pair of dark lateral bands and Ψ-shaped markings behind posterior eyes, markings starting from behind PME and PLE almost reaching dark fovea. Fovea longitudinal. In dorsal view, AER recurved and slightly narrowed than procurved PER. Eye diameters and interdistances: AME 0.16, ALE 0.18, PME 0.16, PLE 0.15; AME–AME 0.14, AME–ALE 0.19, PME–PME 0.38, PME–PLE 0.32. MOQL 0.51, MOQA 0.46, MOQP 0.72. *Chelicerae* protruding and coloured as carapace, three promarginal teeth and two retromarginal teeth. Endites brown, longer than wide. Labium dark brown, longer than wide. Sternum 2.10 long, 1.45 wide. *Abdomen* oval, brown, with conspicuous anterior tufts of hairs, dorsum with dense grey hairs and two pairs of muscle impression, and with broken dark median band, reaching half of opisthosoma length, posteriorly with paired dark markings consisting of numerous stripes and spots; venter brown. *Legs* brownish yellow, all legs with conspicuous dark brown annuli in the distal parts of femur, patella, tibia, metatarsus and tarsus. Measurements of legs: I 8.60 (2.52, 3.20, 1.70, 1.20), II 9.07 (2.64, 3.46, 2.00, 0.97), III 7.49 (2.20, 2.40, 2.06, 0.83), IV 10.43 (2.86, 3.57, 2.82, 1.19).

*Palp* (Figs 4–6, 15–17). RTA dark, small but strong, triangular; cymbium longer than wide, bulb nearly spherical and proapically membranous; sperm duct distinct and sinuate, U-shaped or reversed S-shaped; embolus slender and filiform, originated at 8–9 o’ clock position in prolateral view, its tip slightly overpasses the genital bulb; conductor with a heavily sclerotized and beak-shaped apex, its base part membranous and crane’s neck-shaped; tegular apophysis small and petal-shaped in retrolateral view.

***Female***. Total length 6.53–7.83. One paratype (Fig. 2) measured, body 7.70 long; carapace 3.03 long, 2.08 wide; abdomen 4.55 long, 2.41 wide. Eye sizes and interdistances: AME 0.13, ALE 0.14, PME 0.15, PLE 0.12; AME–AME 0.14, AME–ALE 0.16, PME–PME 0.34, PME–PLE 0.27. MOQL 0.49, MOQL 0.42, MOQP 0.66. Sternum 1.71 long, 1.18 wide. Measurements of legs: I 6.40 (1.83, 2.36, 1.24, 0.97), II 7.04 (2.02, 2.71, 1.41, 0.89), III 5.02 (1.60, 1.83,1.33, 0.27), IV 8.43 (2.32, 2.82, 2.44, 0.86). General characters as in male, but slightly larger in size and darker in colour.

*Epigyne* (Figs 13–14, 18–19). Atrium large and nearly inverted trapezoidal, with a shallow depression, located at anterior portion of epigynal plate, anterior atrial margin “M” shaped; spermathecae and burse are prominently through epigynal plate in ventral view; two copulatory openings located at basolateral atrial borders; spermathecae consisting of papilliform base, tubular stalk and ovoid head, ascend spirally; bursae globular and translucent; fertilization ducts short, acicular.

###### Natural history.

*Clubiona grucollaris* sp. n. mainly inhabit the upper levels of the forest and most specimens were collected by canopy fogging, while a few spiders were obtained by beating twigs and branches of vegetation. The type locality, Fanjing Mountain Nature Reserve, extending from 27°49'50" to 28°01'30"N and 108°49'30"to 108°18'30"E, is the core zone and the highest peak of the Wuling Mountains, and is known for its high floral biodiversity (Wang et al. 2015). The evergreen broad-leaved forests, where the holotype was obtained, are located in low elevation areas (alt. 300–600 m) of the Reserve.

###### Distribution.

**Figure 20.**
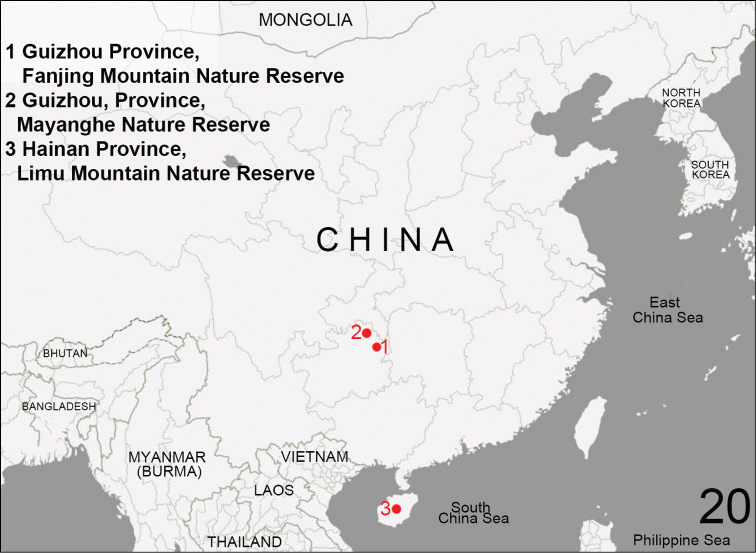
Distribution of *Clubiona grucollaris* sp. n. (red circles).

Guizhou Province (Mt. Fanjing, Mayanghe natural reserves) and Hainan Island (Mt. Limu), China (Fig. 20).

##### Clubiona filicata

Taxon classificationAnimaliaAraneaeClubionidae

O. Pickard-Cambridge, 1874

Figs 3 , 7–8 , 9–12

Clubiona filicata O. P.-Cambridge, 1874: 413, fig. 35 (description of ♂, ♀); Gravely 1931: 261, fig. 16d; Tikader and Biswas 1981: 69, figs 120–121; Gong 1989: 109, figs 1–13; Zhang and Hu 1989: 58, figs 7, 22; Majumder and Tikader 1991: 23, figs 30–35; Biswas and Raychaudhuri 1996: 199, figs 27–33; Song et al. 1999: 415, figs 245L–M, 248F–G; Dankittipakul and Singtripop 2008: 37, figs 5–7, 30–33; Dankittipakul et al. 2012: 59, figs 25–31; Yin et al. 2012: 1095, figs 575a–e.
Clubiona distincta Thorell, 1887: 48
Clubiona swatowensis Strand, 1907: 562 (Description of ♀); Strand 1909: 39, fig. 24.

###### Examined material.

1 ♂, China, Guangxi Province, Guilin City, Guilin Tea Science and Research Institute (150 m; 27°17'48"N, 110°21'34"E), 3 October 2010.

###### Description.

***Male*** (Figs 3, 7–12). For details see Dankittipakul and Singtripop (2008).

###### Natural history.

The examined specimen was collected by a pitfall trap set in a tea plantation.

###### Distribution.

India, Bangladesh, Pakistan, Thailand, Myanmar, Laos, China (see Table 1).

## Supplementary Material

XML Treatment for japonica

XML Treatment for Clubiona grucollaris

XML Treatment for Clubiona filicata
